# Role of Multiphasic Computed Tomography in the Evaluation of Neoplastic Pancreatic Masses: A Single-Center Observational Study

**DOI:** 10.7759/cureus.83606

**Published:** 2025-05-06

**Authors:** Prajwal TR, Umakant Prasad, Deepak Kumar, Neetu Sinha, Rashmi R Bharti, Rakesh Kumar Singh, Ankita Kumari

**Affiliations:** 1 Radiodiagnosis, Indira Gandhi Institute of Medical Sciences, Patna, IND; 2 Pathology, Indira Gandhi Institute of Medical Sciences, Patna, IND; 3 Surgical Gastroenterology and Liver Transplant, Indira Gandhi Institute of Medical Sciences, Patna, IND

**Keywords:** common bile duct, lymph node, main pancreatic duct, metastasis, multiphasic computed tomography, pancreatic adenocarcinoma, superior mesenteric artery, superior mesenteric vein

## Abstract

Background: Pancreatic neoplasms are associated with high morbidity and mortality, largely due to late diagnosis and challenges in accurate staging. Multiphasic computed tomography (CT) is a critical imaging tool for evaluating pancreatic masses offering detailed information on lesion characterization and surgical resectability. Precise radiological assessment is vital for treatment planning, and correlating imaging findings with histopathology improves diagnostic efficiency.

Methods: This prospective study was conducted on 50 patients with clinically suspected pancreatic neoplasms who underwent multiphasic CT imaging. Non-contrast, late arterial (pancreatic phase), and portal venous phase images were acquired following standardized contrast-enhanced CT protocols. Lesions were evaluated for size, morphology, enhancement patterns, vascular involvement, and local invasion. Resectability was assessed according to the Dutch Pancreatic Cancer Group (DPCG) criteria. Imaging findings were correlated with the final histopathological diagnosis to determine diagnostic sensitivity and specificity.

Results: In the vast majority of cases, 43 out of 50 (86%) were diagnosed as adenocarcinoma. Other diagnoses, such as mucinous cystadenoma, solid pseudo-papillary epithelial neoplasm (SPEN), and neuroendocrine tumor, were very uncommon, each making up 4% of the total cases. Multiphasic CT showed excellent sensitivity and specificity of 100% and 85.7%, respectively, in diagnosing pancreatic neoplasms. Out of 50 cases, 16 cases were found to be resectable, 10 cases were borderline resectable, and 24 cases were non-resectable, out of which 15 cases showed distant metastasis.

Conclusion: Multiphasic CT is a highly accurate, non-invasive modality for the evaluation and characterization of pancreatic masses. It demonstrates a strong correlation with histopathological findings, reliably assesses vascular involvement, and predicts resectability according to DPCG criteria. Therefore, early and accurate CT-based evaluation is critical for optimal treatment planning and improving patient outcomes.

## Introduction

Pancreatic cancer typically affects elderly patients and is rarely diagnosed before the age of 30. About 90% of the newly diagnosed patients are aged over 55 years [1]. Pancreatic cancer has a low five-year survival rate, due to delayed diagnosis and low resection rates at presentation. Pancreatic masses present significant diagnostic and management challenges due to their complex nature and the crucial anatomical positioning of the pancreas. The principal modality for imaging pancreatic carcinoma is multiphasic contrast-enhanced computed tomography (CECT) due to its ability to correctly define the extent of tumor infiltration and the existence of metastases which is crucial for accurate diagnosis, staging, and planning of treatment.

Multiphasic computed tomography (CT) plays a crucial role in determining the resectability of adenocarcinoma [2]. The efficacy of multiphasic CT in identifying and characterizing pancreatic masses has been substantiated through various studies that highlight its superior contrast resolution and ability to capture dynamic vascular enhancements. Multiphasic CT aids in determining the resectability of tumors, a crucial factor in patient prognosis. Furthermore, the application of multiphasic CT extends beyond malignant conditions, aiding in the diagnosis of benign pancreatic diseases such as cysts and neuroendocrine tumors, which require different therapeutic approaches. By analyzing enhancement patterns and the level of vascular involvement, multiphasic CT may distinguish pancreatic adenocarcinoma from other pancreatic diseases including neuroendocrine tumors or localized pancreatitis, as reported by Elbanna et al. [2]. Because of their abundant blood supply, hypervascular lesions, including neuroendocrine tumors, stand out during the arterial phase, in contrast to the hypovascular appearance of adenocarcinomas.

The ability to visualize organs in multiple vascular phases not only enhances the detection and characterization of lesions but also significantly impacts clinical outcomes and allows healthcare providers to make more informed decisions regarding biopsy, surgery, and other therapeutic interventions. Additionally, this imaging modality plays a critical role in monitoring treatment responses and detecting recurrence. The aim of this study was to study the role of multiphasic CT in the detection and evaluation of neoplastic pancreatic masses. It also aims to investigate the utility of CECT in differentiating benign from malignant pancreatic lesions and to assess the resectability of neoplastic pancreatic masses based on CT findings.

## Materials and methods

Study design and patient selection

This study was carried out in 50 patients referred to the Department of Radiodiagnosis (patients referred from the Departments of Gastro-surgery, Gastro-medicine, and Oncology) at Indira Gandhi Institute of Medical Sciences (IGIMS), Sheikhpura, Patna, for CECT scan of the abdomen for suspected pancreatic masses. After receiving clearance from IGIMS' Institutional Ethics Committee (approval number: 625/IEC/IGIMS/2022), the study proceeded for two years, from July 2022 to July 2024. All the patients clinically suspected to have pancreatic masses or those in whom previous imaging studies showed a pancreatic mass were included in the study. The study excluded patients in whom contrast agents were contraindicated (e.g., chronic kidney disease (serum creatinine >1.5), advanced diabetes mellitus, single functioning kidney), patients with peri-ampullary masses, pregnant women, patients with a history of contrast agent allergy or anaphylaxis, and patients who did not provide valid consent.

Multiphasic CT pancreatic protocol

Using a Toshiba Aquilion CXL 128-slice multidetector CT (MDCT) equipment (Toshiba Corporation, Tochigi, Japan), CT images of the pancreas were acquired at 120-kV, 500-mAs, 0.5-mm reconstruction interval, 2.5-mm collimation, and 0.5-second gantry rotation time. The contrast agent used was iohexol (Omnipaque 350). The patient was advised to fast for at least six hours before the examination and given a spasmolytic drug (Buscopan 20-40 mg), at the beginning of the examination by intravenous (IV) route or 10-15 minutes before examination by intramuscular (IM) route, which was administered to dilate the duodenum and to decrease the peristaltic movement of the stomach and duodenum. Twenty to 30 minutes prior to the scan, 800 ml of water was taken orally. Iohexol (1-1.5 ml/kg body weight) was administered intravenously at a rate of 3-5 ml/s along with 30 ml of saline chaser. The arterial phase/pancreatic phase and portal venous phase were acquired after 35-40 s and 65-70 s of contrast injection, respectively.

Patient assessment and evaluation

The images were interpreted using multiplanar reconstruction (MPR) in axial, sagittal, and coronal planes. Age, gender, morphological distribution (solid/cystic/mixed), location distribution (head/neck/body/tail/uncinate process), common bile duct (CBD) status, main pancreatic duct (MPD) status, stent in situ (endoscopic retrograde cholangiopancreatography (ERCP)/percutaneous transhepatic biliary drainage (PTBD)), arterial involvement (superior mesenteric artery (SMA)/celiac artery (CA)/common hepatic artery (CHA)/splenic artery (SA)), venous involvement (superior mesenteric vein (SMV)/main portal vein (MPV)/splenic vein (SV)), aberrant hepatic arterial anatomy based on Michel's classification, surrounding infiltration, lymph node involvement, distant metastasis, and resectability based on the Dutch Pancreatic Cancer Group (DPCG) criteria were all assessed in CT images of neoplastic pancreatic masses. The study subjects were then classified into resectable, borderline resectable, and unresectable according to DPCG criteria (Table 1) [3].

**Table 1:** DPCG resectability criteria DPCG: Dutch Pancreatic Cancer Group; CA: celiac artery; SMA: superior mesenteric artery; CHA: common hepatic artery; SMV: superior mesenteric vein; PV: portal vein Reference: [3]

Vessel involved	Resectable	Borderline resectable	Unresectable
Degree of contact with arteries: CA, SMA, or CHA	No contact	≤90°	>90°
Degree of contact with veins: SMV and PV	≤90°	90-270°	>270° or occlusion

Aberrant anatomy of hepatic arteries

The classical form of CHA originating from the CA, continuing as a proper hepatic artery, and dividing into the right hepatic artery (RHA) and left hepatic artery (LHA) to supply the right and left lobes of the liver is not seen in all cases. The hepatic arterial system of the study subjects was categorized according to Michel's classification [4].

Loco-regional lymph nodes and extra-regional lymph nodes

Extra-regional lymph nodes in pancreatic carcinoma are para-aortic lymph nodes and lymph nodes that are on the left side of SMA. A lymph node is considered enlarged if its short-axis diameter is greater than 10 mm. Other criteria include central necrosis, heterogeneity, and rounded shape, among others.

CBD was considered dilated if its maximal intraluminal diameter was greater than 7 mm. MPD was regarded as dilated if its maximal intraluminal diameter in the head area was greater than 2.5 mm.

Statistical analysis

The collected data were analyzed statistically by IBM SPSS Statistics for Windows, V. 21.0 (IBM Corp., Armonk, NY, USA), to draw inferences. For quantitative data, descriptive statistics like mean and range were employed, and for qualitative (categorical) data, frequency, percentage (%), and the chi-squared test were used to describe the distribution of categories. An independent t-test was used for continuous variables. The receiver operating characteristic (ROC) curve was plotted to evaluate the diagnostic performance. A p-value of less than 0.05 was considered significant.

## Results

Demographic distribution

Most of the patients, representing 42% (21), were in the 50-69-year age group. The youngest age group, 10-29 years, accounted for 12% (six) of the participants, while the oldest age group, 70-90 years, made up the remaining 8% (four) (Table 2). Around 56% (28) of the study subjects were females, and the rest (44%, 22) were found to be males.

**Table 2:** Age distribution of patients with pancreatic neoplasm

Range of age (in years)	No. of patients	Percentage (%)
10-29	6	12
30-49	19	38
50-69	21	42
70-90	4	8
Total	50	100

Imaging characteristics of pancreatic mass

**Figure 1:**
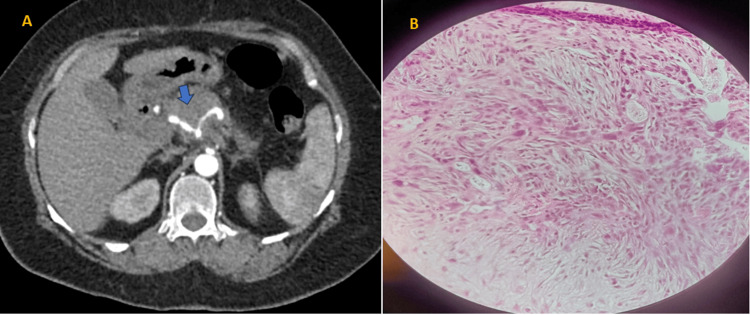
(A) Contrast-enhanced CT scan image of the abdomen in the arterial phase shows a mildly enhancing solid mass in the head of the pancreas encasing the celiac trunk, splenic artery, and common hepatic artery (precontrast HU-47, postcontrast HU-65). (B) Microscopic section of the pancreas shows infiltrating well- to poorly formed glandular/ductal structures surrounded by remarkably desmoplastic stroma (H&E stain, original magnification 10×). It was conformed to be a case of adenocarcinoma of the head of the pancreas CT: computed tomography; H&E: hematoxylin and eosin; HU: Hounsfield units

The largest percentage of pancreatic masses were solid (66%, 33) (Figure 1A).

**Figure 2:**
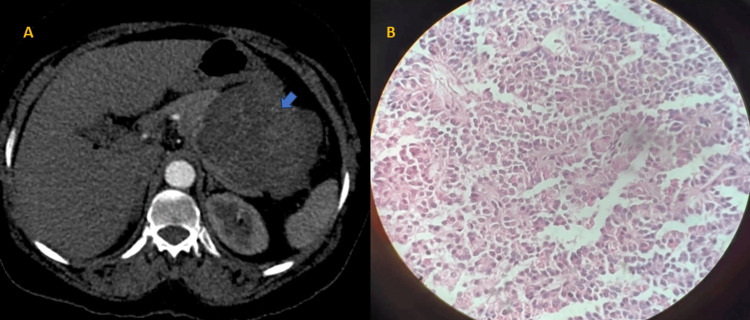
(A) Axial contrast-enhanced CT scan image of the abdomen in portal venous phase at the level of the pancreas shows a well-defined solid cystic mass in the body and tail of the pancreas. (B) Microscopic section of the pancreas shows tumor cells arranged in solid and pseudo-papillary pattern, solid areas comprising monomorphic cells admixed with capillary-sized blood vessels, and pseudo-papillae formed by tumor cells around the blood vessels, getting detached at places (H&E stain, original magnification 20×). It was proven to be a case of SPEN CT: computed tomography; H&E: hematoxylin and eosin; SPEN: solid pseudo-papillary epithelial neoplasm

The second highest percentage were mixed masses (30%, 15) (Figure 2A).

**Figure 3:**
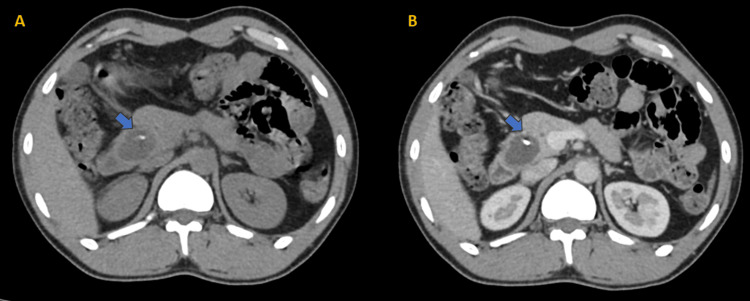
Plain (A) and portal venous phase of contrast-enhanced (B) axial CT scan images show a thick-walled hypodense cystic lesion with tiny focus of calcification at the periphery. On HPE, it was proven to be a case of mucinous cystadenoma of the pancreas CT: computed tomography; HPE: histopathological examination

**Figure 4:**
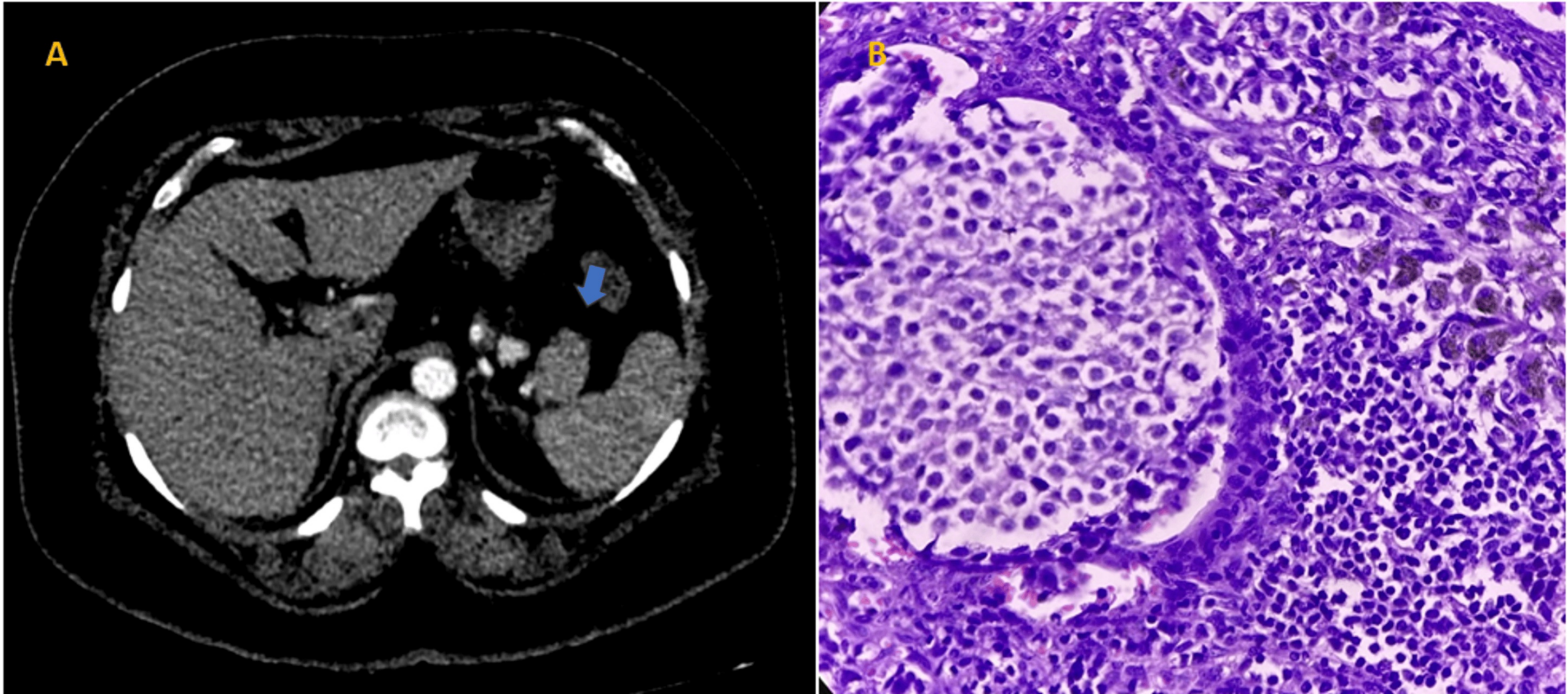
(A) Axial contrast-enhanced CT scan image in arterial phase demonstrates small hyperenhancing lesion in the tail of the pancreas. (B) Solid nests, trabeculae, and cords of tumor cells having eosinophilic to amphophilic, finely granular cytoplasm and monomorphic centrally located round to oval nuclei with salt and pepper appearance (H&E stain, original magnification 40×). It was determined to be a case of insulinoma CT: computed tomography; H&E: hematoxylin and eosin

Only a small fraction of the masses was purely cystic (4%, two). In 22 cases (44%), neoplastic pancreatic mass involved the head of the pancreas, making it the most frequent location. About 52% (26) of pancreatic masses were large-sized (>4 cm), accounting for more than half of the total. The greatest number of pancreatic masses were large-sized (>4 cm) constituting 52% (26), followed by the 2-4-cm range which constitutes 46% (23) and <2-cm range making the rest 2% (one) of the masses. The majority of pancreatic masses, accounting for 60% (30) of the cases, were found to be hypovascular with mild postcontrast enhancement (pre- and postcontrast difference of 10-15 Hounsfield units (HU)) (Figure 1A). Apart from this, 4% (two) of the masses showed arterial phase hyperenhancement, and 4% (two) were purely cystic showing wall enhancement (Table 3, Figure 3A, and Figure 4A).

**Table 3:** Imaging features of pancreatic neoplastic masses on CT scan CT: computed tomography; IPMN: intra-ductal papillary mucinous neoplasm; SPEN: solid pseudo-papillary epithelial neoplasm; CBD: common bile duct; MPD: main pancreatic duct

Lesion	Typical location	Non-contrast CT	Enhancement pattern	Margins	Calcifications	Ductal involvement
Adenocarcinoma	Head	Hypodense	Hypovascular, mild enhancement	Ill defined, infiltrative	Rare, small, punctate	Double duct sign (dilated both CBD and MPD)
Neuroendocrine tumor	Anywhere	Iso- to hypodense	Arterial phase hyperenhancement	Well defined	Rare	Usually no
Serous cystadenoma	Body/tail	Hypodense	Septal enhancement	Well defined	Central stellate scar may calcify	No
Mucinous cystadenoma	Body/tail	Hypodense	Wall/septal enhancement	Well defined	Peripheral rim-like calcification	May compress the duct (MPD)
IPMN	Head-main duct IPMN, uncinate-side branch IPMN	Cystic/dilated duct appearance	Wall/septal enhancement	Segmental or diffuse ductal dilatation	May have coarse calcification	MPD dilatation
SPEN	Tail>body	Heterogeneous	Heterogeneous enhancement of solid components	Well encapsulated	Rare	Rare

Status of CBD and MPD

The analysis revealed that most of the cases, 36 out of 50, showed no dilatation of the CBD, representing 72% of the total. Conversely, CBD dilatation was observed in 14 cases, accounting for 28% of the total. MPD involvement was observed in 15 cases, accounting for 30% of the total. Thirty-five out of 50 cases did not involve the MPD, representing 70% of the total. Only 4% (two) of the cases showed PTBD stent in situ.

Arterial and venous involvement

**Figure 5:**
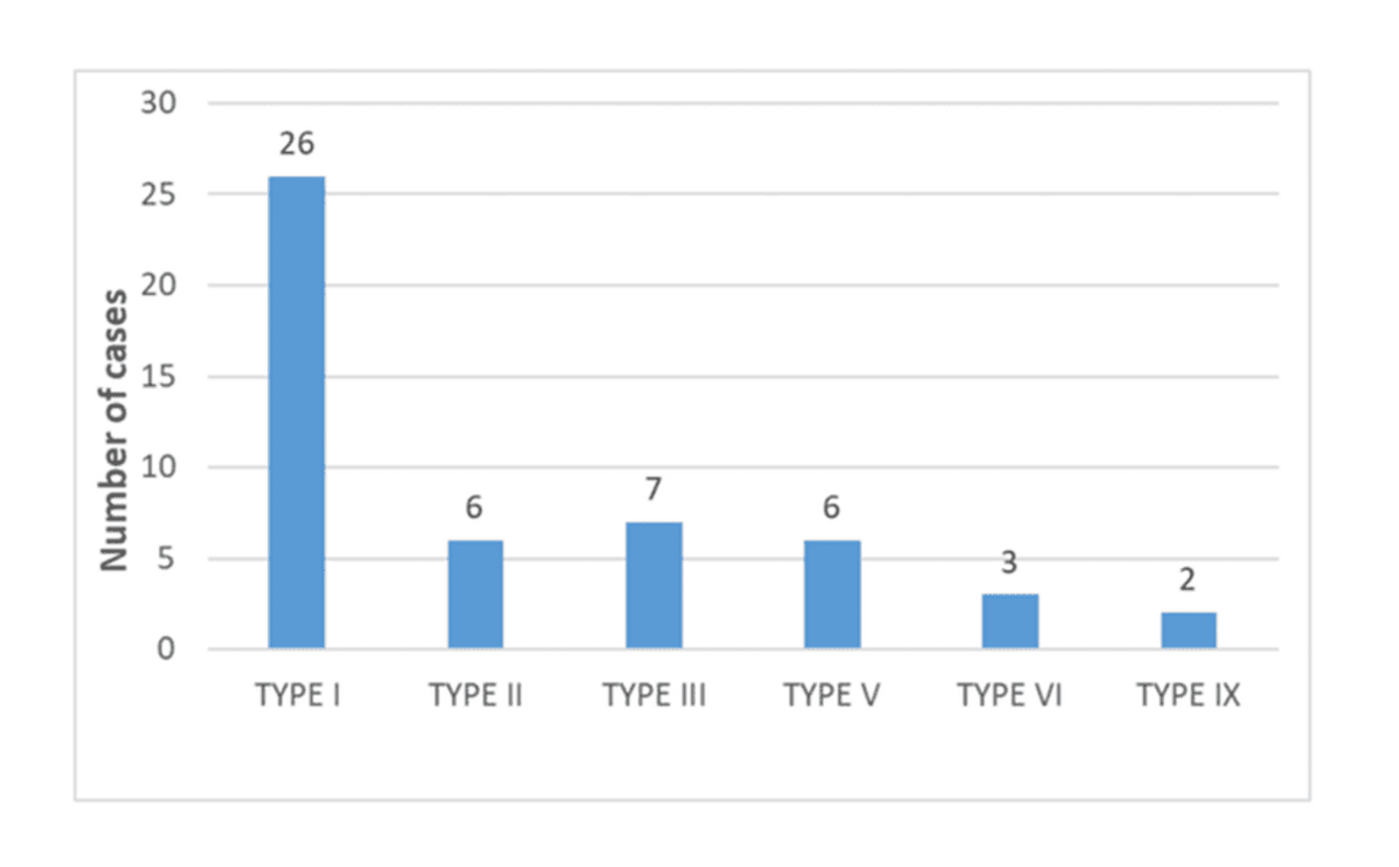
Histogram showing the distribution of aberrant artery anatomy in patient with pancreatic mass

Out of 50 cases, 56% (28) cases showed various degrees of arterial involvement. Among the cases, 28% (14) had CHA involvement and 26% (13) had SMA involvement. CA involvement was seen in 26% (13) of the cases, while SA involvement was present in 16% (eight) of the cases (Figure 1). Around 66% (33) of the cases showed various degrees of venous involvement (abutment/encasement). Venous thrombosis was noted in one case (2%). About 26% (13) of the cases showed SV involvement, 54% (27) of the cases showed SMV involvement, and 34%(17) of the cases showed MPV involvement. The analysis revealed that type I (standard anatomy) aberrant anatomy of hepatic arteries was the most prevalent anatomical pattern, observed in 26 out of 50 cases, accounting for 52% of the total (Figure 5).

Lymph node involvement, surrounding tissue infiltration, and distant metastasis

The study revealed that 40 out of 50 cases, or 80% of the total, had lymph node involvement. Extra-regional lymph nodes were seen in 20 (40%) of the 40 cases with lymph node involvement. A significant number of cases 70% (35) exhibited surrounding tissue infiltration. The first and second parts of the duodenum and the pylorus of the stomach were the most common sites of infiltration. Approximately 30% (15) of cases showed distant metastasis. Twelve patients (24%) had liver metastases, six cases (12%) had omental metastases, four cases (8%) had lung metastases, and three cases (6%) had spinal metastases. The liver was the most common organ of metastasis.

Distribution of pancreatic masses based on histopathological examination (HPE)

Out of 50 cases, 43 (86%) were found to be malignant, possibly adenocarcinoma. Forty (80%) of these cases had histological confirmation of adenocarcinoma, and one was found to have mass-forming chronic pancreatitis (Figure 1B and Table 4). The latter displayed a poorly defined hypoattenuating mass that was hypoenhanced in comparison to the remaining pancreatic parenchyma. Chronic pancreatitis with mass formation was additionally favored by calcifications within MPD and parenchyma, as well as peripancreatic fat streaking. Malignancy is favored by vascular encasement and the double duct sign.

**Table 4:** Distribution based on the HPE diagnosis of pancreatic masses HPE: histopathological examination; NET: neuroendocrine tumor; SPEN: solid pseudo-papillary epithelial neoplasm

HPE diagnosis	Frequency	Percent
Adenocarcinoma	40	80
Mucinous cystadenocarcinoma	1	2
Mucinous cystadenoma	2	4
NET	2	4
SPEN	2	4
Cases with unknown HPE status	2	4
Alternative diagnosis on HPE	1	2
Total	50	100

Two cases (4%) that had been identified on CT as solid pseudo-papillary epithelial neoplasm (SPEN) were also verified on HPE (Figure 2B). Another two cases (4%) were diagnosed as mucinous cystadenoma on CT and confirmed by HPE (Figure 3B). The HPE status of the other two cases was unknown. Two cases (4%) were diagnosed as neuroendocrine tumors on CT, both confirmed as functional insulinomas on HPE (Figure 4B and Table 5).

**Table 5:** Comparison between the CT diagnosis and HPE diagnosis CT: computed tomography; HPE: histopathological examination

Type of tumor	No. of cases diagnosed on CT	No. of cases confirmed on HPE	No. of cases with alternative diagnosis on HPE	No. of cases with unknown HPE status
Malignant mass possibly adenocarcinoma	43	40	1 (mass-forming chronic pancreatitis)	2
Neuroendocrine tumor	2	2	0	0
Mucinous cystadenoma	2	2	0	0
Mucinous cystadenocarcinoma	1	1	0	0
Solid pseudo-papillary epithelial neoplasm	2	2	0	0

Accuracy of CT in diagnosing pancreatic mass

Forty (80%) of the 43 cases of malignant tumors, possibly adenocarcinoma, identified by CT were confirmed by histological analysis (Table 2). The sensitivity and specificity of multiphasic CT in diagnosing neoplastic pancreatic masses were found to be 100% (CI: 91.2-100%) and 85.7% (CI: 52.9-97.8%), respectively. The p-value of the study was found to be 4.84×10−9((<0.05).

Distribution of pancreatic masses according to resectability

**Figure 6:**
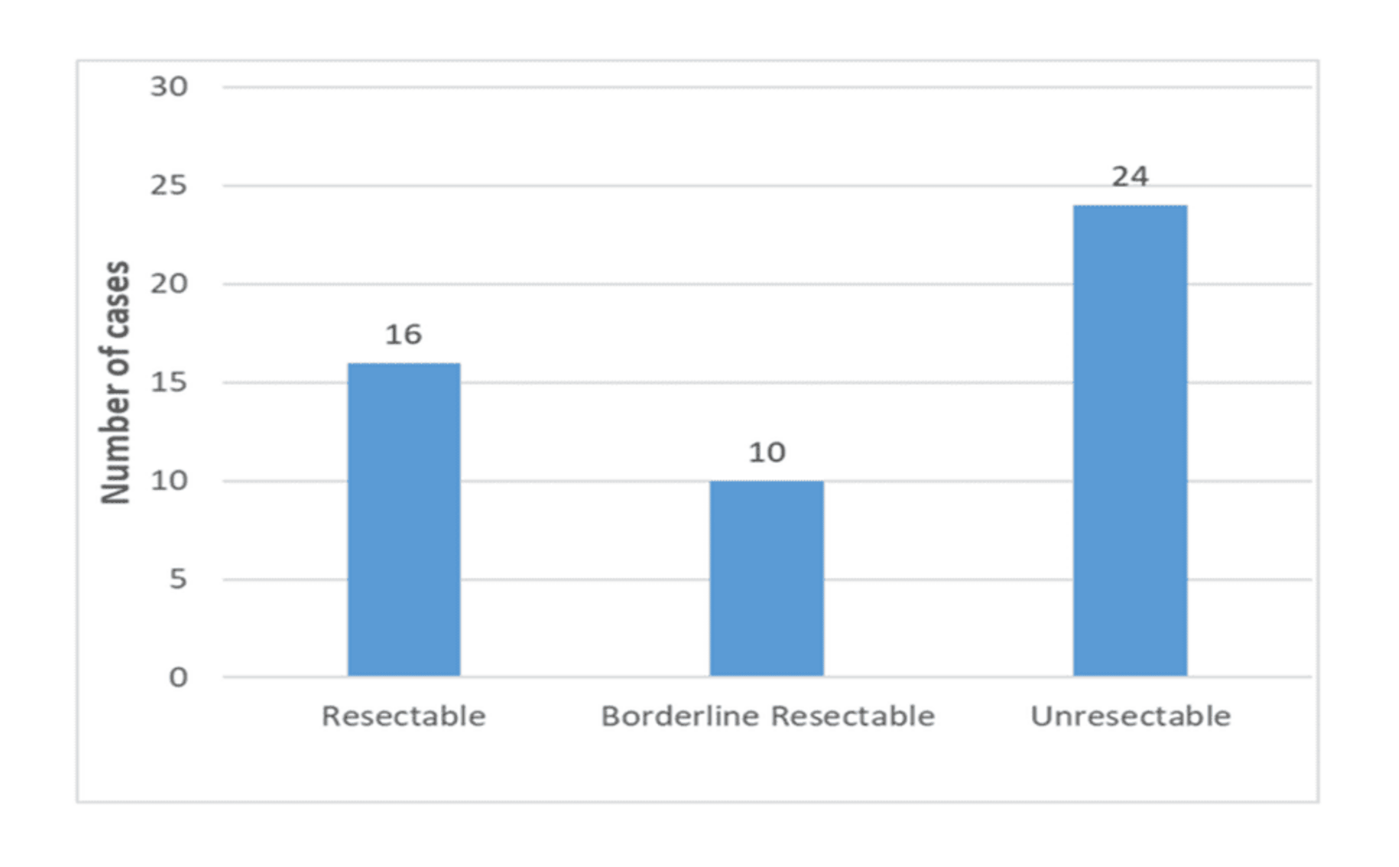
Histogram showing the frequency distribution of pancreatic mass resectability in patients with pancreatic mass

Out of 50 cases, 16 (32%) were found to be resectable, 10 (20%) were borderline resectable, and 24 (48%) were unresectable, out of which 15 (30%) showed distant metastasis (Figure 6).

## Discussion

In the present study, pancreatic masses were analyzed under various parameters like vascular involvement, distant metastasis, etc., and the diagnosis was correlated with the histopathological findings. The 50-69-year age group has the highest number (42%) with a mean age of 59.5 years, followed by the 30-49-year age group (38%). This distribution is similar to the studies conducted by Dawoud et al. [5] and Azzaz et al. [6], which also observed a higher prevalence of patients within the middle- and old-aged demographic. Like our study, Azzaz et al. [6] and Gulve et al. [7] highlighted varied distribution across different age groups for both genders and observed a predominance of female participants. This trend underscores the need for further investigation into the factors contributing to higher female participation in health-related studies. However, it is important to note that the sample size is relatively small and larger studies would be needed to draw more definitive conclusions about gender differences in the prevalence of pancreatic masses.

A significant portion of the masses in our investigation consisted of solid masses (38%) and mixed masses (58%). Polk et al. [8] demonstrated a preponderance of mixed masses in their investigation also. The predominance of mixed and solid masses suggests that many of the pancreatic masses in this study were likely to be malignant, as solid morphology is often associated with malignant lesions. The most frequent location for pancreatic masses was the head of the pancreas. A sensitivity of 87.5% was attained in the detection of pancreatic masses in the studies by Dawoud et al. [5], Azzaz et al. [6], and Hossain et al. [9], which also reported a higher prevalence of pancreatic masses in the head of the pancreas. These consistent results highlight the significance of the pancreatic head as a critical area for the occurrence of masses, emphasizing the need for focused diagnostic and therapeutic approaches in this region.

Most of the pancreatic masses were greater than 4 cm (52%) in our study. This indicates that more than half of the patients had large pancreatic masses. Larger pancreatic masses were also more common, according to several investigations done by Dawoud et al. [5], Hossain et al. [9], and Almeida et al. [10]. These reliable findings emphasize how crucial it is to identify and treat pancreatic masses early to improve patient outcomes.

Similar to our results, research by Dawoud et al. [5], Azzaz et al. [6], and Chen et al. [11] showed mild enhancement patterns in the majority of the pancreatic masses. This predominant mild enhancement pattern suggests that most of the masses have a relatively low degree of vascularity, which is a common feature in various types of pancreatic neoplasms including the most common adenocarcinoma. Intense enhancement was noted in two cases in our study, and they all were neuroendocrine tumors. Understanding these enhancement patterns is crucial for radiologists and clinicians in assessing the vascularity and potential malignancy of pancreatic masses, thereby guiding appropriate diagnostic and therapeutic strategies.

The analysis revealed that the majority of cases, 36 out of 50, showed no dilatation of the CBD, representing 72% of the total. Conversely, CBD dilatation was observed in 14 cases, accounting for 28% of the total. Hossain et al. [9] in their study reported CBD dilatation in 45% of the study subjects, and in the study conducted by Singhal et al. [12], isolated CBD dilatation was observed in two cases (8%). The presence of CBD involvement suggests that these pancreatic masses either were located in close proximity to the duct or were large enough to exert pressure on it, leading to possible obstruction. Patients without CBD involvement may have different treatment options compared to those with involvement, who might need more immediate intervention to address biliary obstruction and associated complications.

MPD involvement was observed in 30% of the cases in our study. Several studies by Singhal et al. [12], Hossain et al. [9], and Dawoud et al. [5] also revealed comparable patterns of MPD involvement. This underscores the importance of recognizing MPD dilatation in pancreatic mass assessments. The presence of MPD involvement in these cases suggests that the pancreatic masses had extended to or were compressing the MPD, which can lead to significant clinical symptoms such as obstructive jaundice, abdominal pain, and pancreatitis. MPD involvement often complicates the management of pancreatic masses, requiring careful surgical planning and possibly additional interventions to address ductal obstruction.

Stent placement was present in only two cases. The presence of a stent in these patients indicates that their pancreatic masses caused sufficient obstruction to warrant this intervention. Studies performed by Azzaz et al. [6], Wong and Raman [13], and Gerritsen et al. [14] also reported low rates of stent placement due to obstructive pancreatic masses. However, stent placement is a necessary intervention in cases of severe obstruction caused by pancreatic masses.

Arterial involvement such as SMA, CHA, CA, and SA is a critical factor in assessing the resectability and overall prognosis of pancreatic masses. Less than 180-degree contact is termed abutment, while more than 180-degree contact is termed encasement. Out of 50 cases, 28 showed various degrees of arterial involvement. Azzaz et al. [6] and Gerritsen et al. [14] highlighted the importance of arterial involvement in determining treatment approaches, and the study by Ramaekers et al. [15] found that perivascular CT radiomic features enhance the preoperative assessment of arterial involvement in patients with surgically proven pancreatic ductal adenocarcinoma (PDAC). This advancement aids in better surgical planning and outcomes.

In cases with SMA involvement, six masses (12%) exhibited less than 90 degrees of involvement, three cases (6%) involved 90-270 degrees of involvement, and four cases (8%) had greater than 270 degrees of involvement. This distribution is consistent with previous studies by Dawoud et al. [5] and Hossain et al. [9], supporting the patterns observed in our research regarding the extent of SMA involvement in similar patient populations, and the study conducted by Azzaz et al. [6] observed that the SMV were involved by less than 180° without any distortion or thrombosis. No vascular involvement is a positive indicator for surgical resectability, as the absence of vascular involvement generally makes the surgical removal of the tumor more feasible. The extensive encasement typically indicates a more advanced and aggressive disease, often precluding surgical resection due to the high risk of complications and the difficulty in achieving clear margins without damaging the artery.

According to numerous publications, including Brook et al. [16], Shrikhande et al. [17], and Ahmed Abdallah et al. [18], 45% of the population exhibits variability in hepatic arterial structure. Replaced RHA and replaced LHA are the most common variants. Similarly, in our study, also types II, III, and V were seen in a substantial number of cases. The presence of variant arterial anatomy may complicate surgery; hence, knowledge of common visceral arterial variants is important for accurate preoperative planning.

The study conducted by Singhal et al. [12] demonstrated an accuracy of 94.11% in assessing infiltration, with infiltration observed in 50% of patients highlighting a similar pattern of infiltration in these anatomical regions. The high percentage of surrounding infiltration in this study (70%) indicates that a significant proportion of pancreatic masses extended beyond the primary tumor site into the surrounding tissues. This extensive infiltration is often associated with more advanced disease, complicating surgical resection and potentially requiring more aggressive and comprehensive treatment strategies.

A substantial majority of the cases (80%) exhibited lymph node involvement. Twenty cases showed extra-regional lymph nodes. High rates of lymph node involvement and significant cases of extra-regional lymph node involvement were documented in Singhal et al.'s [12] study, which also reported lymph node involvement in the anterior, posterior, superior, and inferior regions. The predominance of lymph node involvement underscores the aggressive nature of pancreatic masses in this study population, highlighting the importance of thorough lymph node assessment during staging and treatment planning. The presence of metastasis in over a quarter of the cases indicates that a significant portion of the study population had advanced disease.

Anghel et al. [19] and Lee and Lee [20] reported a predominance of adenocarcinoma among their cases, with other tumor types being much less common. In our study, a significant percentage of adenocarcinomas is also observed, which may also be a limiting factor in determining the accuracy of CT scans in other histologic subtypes.

The majority of the cases (24) in our study were unresectable, while 16 were resectable; the result is consistent with studies by Dawoud et al. [5], Azzaz et al. [6], and Hossain et al. [9]. The commonest causes of unresectability in the non-resectable group (24 cases) were vascular invasion, lymph node metastases, and distant metastasis.

Brennan et al. reported that CT has an accuracy of 85-95% for tumor detection, a positive predictive value of 89-100% for unresectability, and a negative predictive value of 45-79% for resectability [21]. Excellent sensitivity of 100% and specificity of 85.7% were demonstrated by our investigation. A considerable proportion of adenocarcinomas were found in the current investigation, which may have contributed to the remarkably high sensitivity. Future research must include a larger sample size and a higher percentage of various histopathological subtypes.

Strengths

Through correlation with histology, the study validates CT findings, enhancing diagnostic reliability. It is useful for researching how CT scans can distinguish between different pancreatic cancer subtypes. It promotes a methodical approach to CT scan reporting, which can significantly help with the treatment of pancreatic cancer.

Limitations

The present study was conducted on a small sample size. Thus, future studies are required to be conducted on a greater number of patients. Magnetic resonance cholangiopancreatography (MRCP), which could have offered important information about pancreatic duct masses, was not carried out in this investigation. There was a lack of intraoperative and postoperative histopathologic information on surgically removed neoplastic pancreatic masses, which would have more accurately verified the tumors' histological state. 

## Conclusions

The study showed that multiphasic CT offers high diagnostic accuracy in the evaluation of neoplastic pancreatic masses providing detailed information on tumor morphology, vascular involvement, metastatic spread, and resectability. These findings underscore the central role of multiphasic CT in diagnostic workflow and treatment stratification in line with DPCG criteria. The modality's ability to facilitate early detection and precise characterization has direct clinical implications, improving short-term outcomes and surgical decision-making. Further prospective studies are warranted to assess the prognostic value of CT and stratification on long-term survival and to refine radiological criteria for future guidance integration.
